# Prognostic Value of Peripheral Blood Lymphocyte/monocyte Ratio in Lymphoma

**DOI:** 10.7150/jca.50552

**Published:** 2021-04-19

**Authors:** Feiqiong Gao, Jianlai Hu, Jiawei Zhang, Yang Xu

**Affiliations:** 1 Department of Hematology, the Second Affiliated Hospital of Zhejiang University School of Medicine, Hangzhou 310009, China.; 2 Department of Prosthodontics, the Second Affiliated Hospital of Zhejiang University School of Medicine, Hangzhou 310009, China.; 3 Zhejiang Provincial Key Laboratory for Cancer Molecular Cell Biology, Life Sciences Institute, Zhejiang University, Hangzhou 310058, China.

**Keywords:** lymphoma, lymphocyte/monocyte ratio, prognosis, overall survival, progression-free survival

## Abstract

**Objective:** Lymphocyte monocyte ratio (LMR) has been considered as a prognostic factor in patients with lymphoma, which focused on diffuse large B-cell lymphoma (DLBCL) and Hodgkin lymphoma (HL). Recently, many relevant clinical studies have been published with inconsistent results. To gain a more comprehensive view of the prognostic value of LMR, we conducted a meta-analysis on the significance of peripheral LMR in all subtypes of lymphoma.

**Methods:** PubMed, PMC, Web of Science, Embase, and Cochrane Library were searched for relevant articles to conduct a meta-analysis. Hazard ratio (HR) and its 95% confidence interval (CI) of OS and PFS were extracted and pooled on stata12.1.

**Results:** In the meta-analysis, forty studies were eligible and a total of 10446 patients were included. Low LMR was associated with an inferior OS (HR=2.45, 95%CI 1.95-3.08) and PFS (HR=2.36, 95%CI 1.94-2.88). In the analysis of lymphoma subtypes, similar results were seen in HL, NHL, and its subtypes including DLBCL, NK/T cell lymphoma, and follicular lymphoma. In addition, low LMR was related with higher LDH (OR=2.26, 95%CI 1.66-3.09), advanced tumor staging (OR=0.41, 95%CI 0.36-0.46), IPI score (OR=0.40, 95%CI 0.33-0.48), but not with bone marrow involvement (OR=1.24, 95%CI 0.85-1.81) or pathological subtype (OR=0.69, 95%CI 0.41-1.16).

**Conclusion:** Low LMR in peripheral blood indicates poor prognosis in patients with lymphoma. As a simple clinical indicator, peripheral blood LMR combined with existing prognostic factors can improve the accuracy of lymphoma prognosis assessment.

## Introduction

Lymphomas are a heterogeneous group of lymphoid malignancy that is classified into Hodgkin Lymphoma (HL) and non-Hodgkin lymphoma (NHL). HL includes classic HL and nodular lymphocyte-predominant HL, and the classic HL can be further divided into four subtypes. Compared with HL, NHL comprises a more complex spectrum of subtypes, 85-90% of which arise from B cells, such as diffuse large B-cell lymphoma (DLBCL), follicular lymphoma (FL), mantle cell lymphoma (MCL) and Burkitt's lymphoma (BL); the remainder derive from T or NK lymphocytes such as NK/T cell lymphoma (NK/TL) and peripheral T cell lymphoma (PTCL) 1.

Advances in target therapy, adoptive cell therapy, and stem cell transplantation have improved the clinical outcomes of patients with lymphoma, however, relapsed or refractory diseases remain significant unmet needs in lymphoma treatment. According to the US data in 2019, about 19970 died of the disease among newly diagnosed 74200 NHL patients 2. Accurate prognostic stratifications are essential for individualized or precision therapy in order to reduce the mortality and improve the quality of life in lymphoma patients. At present, there are many tools to assess the risk of lymphoma, including international prognostic index (IPI), gene expression profiling (GEP), and positron emission tomography-computed tomography (PET-CT). Nevertheless, PET-CT is relatively expensive, GEP analysis is labor-intensive and time-consuming, and IPI does not take into account the patients' immune status and tumor microenvironment (TME). Thus, simple and appropriate immune biomarkers have been explored to better predict the prognosis of lymphoma.

Previous studies have shown that increased tumor-associated macrophages (TAMs) before treatment was associated with poor overall survival in patients with lymphoma 3, 4. Since TAMs are derived from the monocytes in peripheral blood, the number of TAMs is well correlated with that of monocytes. TAM can secrete various cytokines to promote tumor growth as well as angiogenesis in the tumor microenvironment 5. On the other hand, lymphocytes play an important role in immune surveillance. It was reported that absolute lymphocyte count (ALC) was a surrogate marker of immune status, and low ALC was associated with poor prognosis 6, 7. Therefore, peripheral blood lymphocytes to monocytes ratio (LMR) can readily reflect the crosstalk between the patients' immunity and the tumor microenvironment.

The clinical outcomes of many lymphoma subtypes, including FL 8, 9, DLBCL 10, 11, and NK/TL 12, could be predicted by peripheral blood LMR. Due to the heterogeneity of the sample size and the diversity of treatments reported in previous studies, the consistency of the prognostic impact of LMR remains unknown. To clarify the prognostic role of LMR in lymphoma, we conducted a comprehensive meta-analysis to assess the prognostic value of LMR in lymphoma with its subtypes and to reveal the correlation between LMR and clinicopathological characteristics including LDH, pathologies, staging, and IPI score.

## Materials and methods

### Literature search

PubMed, PMC, Web of Science, Embase, and Cochrane Library were searched for relevant studies, with the deadline of February 2020, and the language was restricted to English and Chinese. Search terms included “lymphocyte-to-monocyte ratio” or “lymphocyte monocyte ratio” or “LMR” and “lymphoma”. Two researchers screened the search results according to the inclusion and exclusion criteria. When disagreements occurred, a third reviewer was consulted.

### Inclusion and exclusion criteria

Inclusion criteria were as follows: 1) prospective or retrospective clinical studies; 2) patients diagnosed with lymphoma; 3) reported on the comparison of prognostic value between high LMR and low LMR group; 4) OS (overall survival) or PFS (progression-free survival) should be included; 5) results should be provided in the form of hazard ratio (HR) and 95% confidence interval (CI). Studies were excluded based on the following criteria: 1) animal or cell line experiments; 2) duplicate studies, conference abstracts or those without available full texts; 3) studies that are not related to the research topic or those without relevant results or needed data.

### Data collection and literature quality assessment

The following data were extracted from adopted articles and recorded in a form: first author, publication, country, disease subtypes, sample size, HR and 95%CI of OS and PFS, the use of rituximab or not, LMR cutoff value, and so on. Quality evaluation was conducted independently by two authors based on the Newcastle-Ottawa scale (NOS), according to its three components (selection, comparability, and outcome). Scores ranged from 0 to 9 points, and those with a total score higher than 5 were regarded as high-quality studies.

### Statistical analysis

All analyses were performed using STATA version 12.1 software (StataCorp, College Station, TX, USA). HR and 95% CI were pooled to compare the prognostic significance of LMR in lymphoma on OS and PFS, and results were displayed by forest plots. Subgroup analyses were performed using the same analysis method. Correlation between LMR and clinicopathological parameters of lymphoma were evaluated by OR with its 95%CI. Heterogeneity was checked by the chi-squared test and *I*^2^ statistic (*I*^2^≤ 50%, *P*>0.1 acceptable level of heterogeneity; *I*^2^>50%, *P*≤ 0.1, obvious). Publication bias was assessed by Egger's and Begg's tests. We conducted a sensitivity analysis to estimate whether any single study affected combined HRs. Statistical significance was set at a two-tailed* P*<0.05.

## Results

### Search results and study characteristics

**Figure 1:**
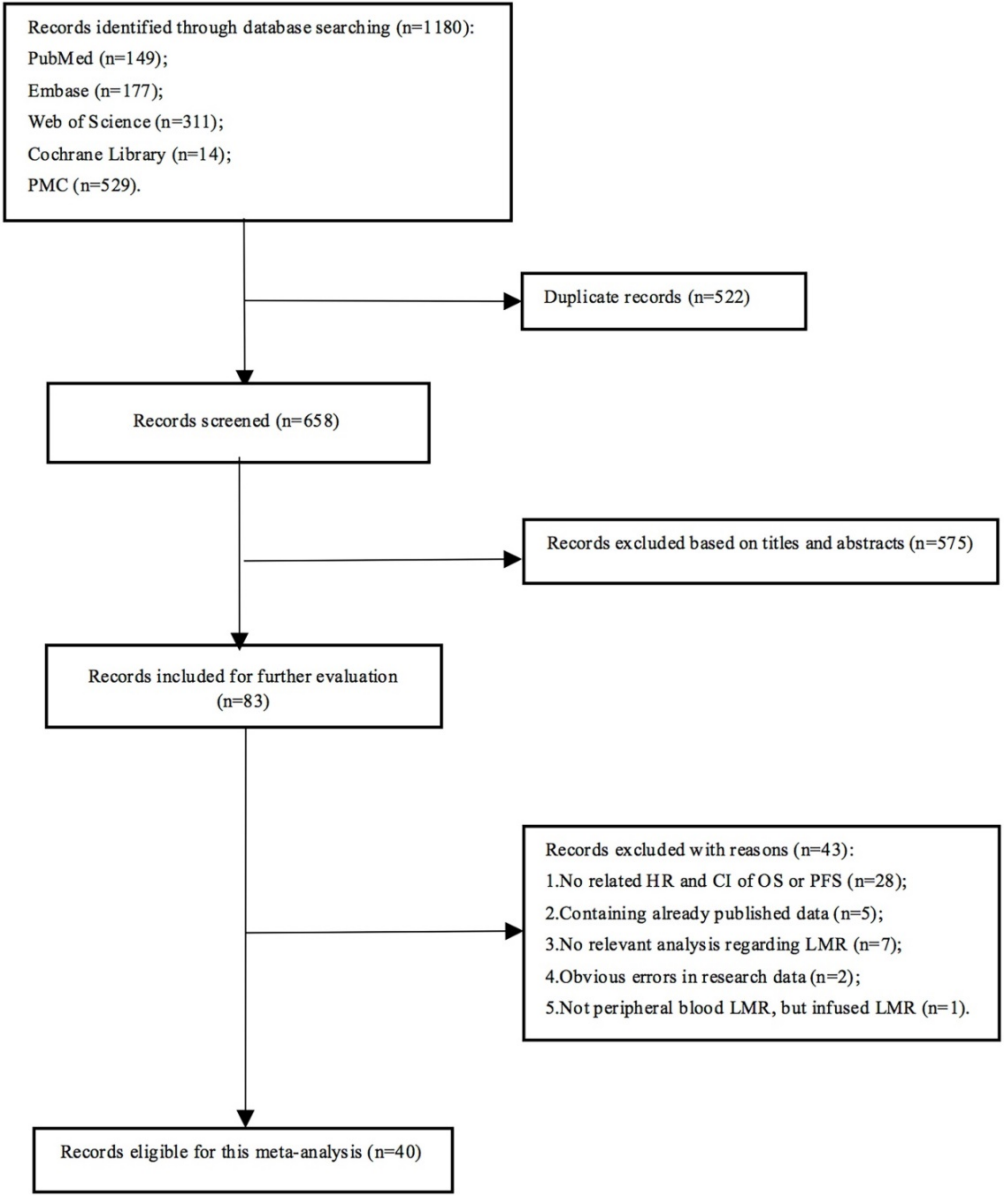
Literature search flow diagram.

A total of 1180 articles were obtained, and 658 were left after 522 duplicates were excluded. After the initial screen, 575 articles were excluded, leaving 83 articles for detailed reading. Finally, 40 articles were eligible for this meta-analysis (Fig. 1), involving 10,446 lymphoma patients. Each study divided patients into high LMR and low LMR groups based on different LMR cut-off values, which were acquired from ROC curves or previous studies or median LMR. Five studies did not present the number of patients in the two groups. In the remaining 35 articles, 3817 patients were assigned to the low LMR group, while 5,082 were included in the high LMR group. All the adopted articles were retrospective studies with NOS scores of 4 or higher. The characteristics of the included studies were shown in Table 1.

### Publication Bias

**Figure 2:**
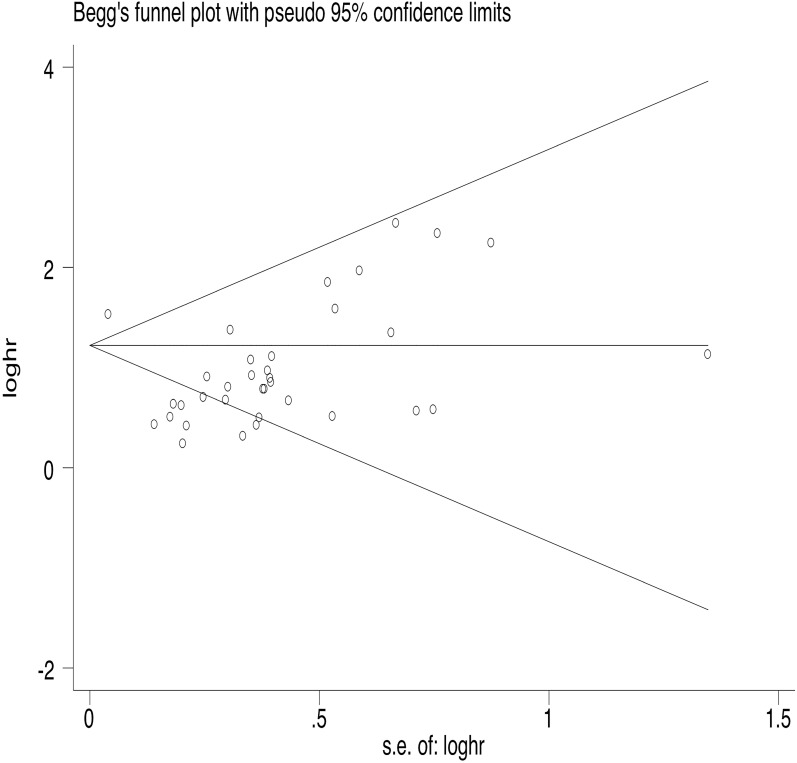
Begg's funnel plot of OS.

**Figure 3:**
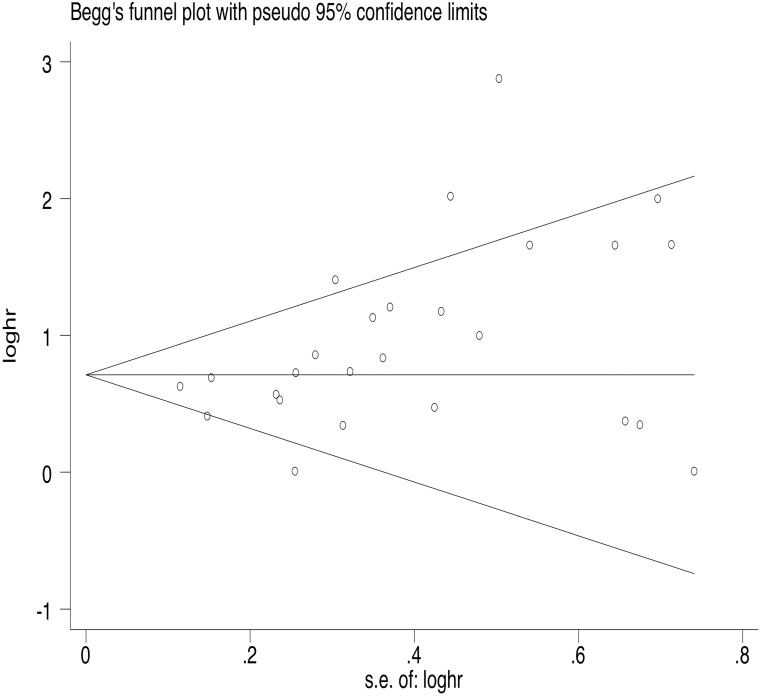
Begg's funnel plot of PFS.

35 articles were available for the analysis for publication bias with regard to the HR of OS. Both Begg's test and Egger's test demonstrated that there was publication bias regarding the HR of OS (Egger's Test: *P*=0.002, Begg's Test: *P*<0.000, Fig. 2). 26 studies reported on PFS. The results also indicated publication bias in PFS, but the bias was not as significant as that of OS (Egger's Test: *P*=0.010, Begg's Test: *P*=0.015, Fig. 3).

### Overall Survival

**Figure 4:**
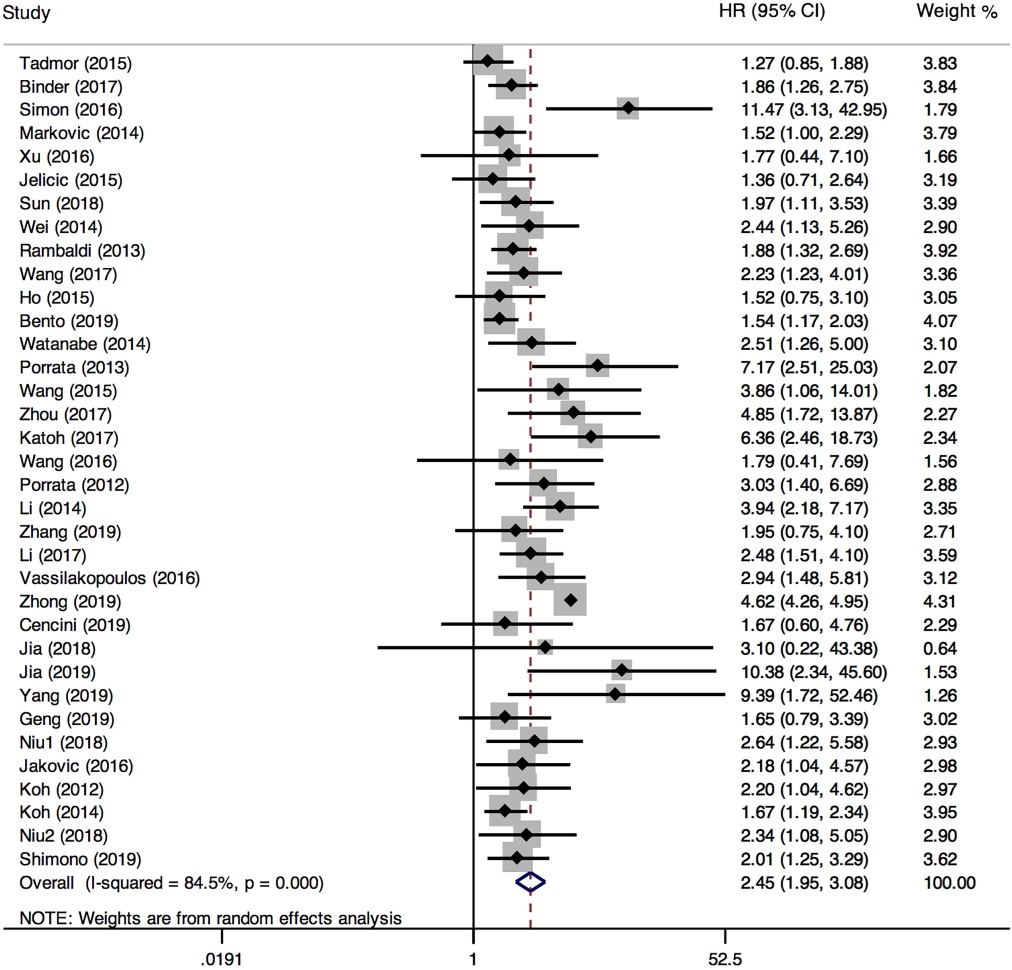
Forest plot comparing OS between low-LMR and high-LMR groups.

**Figure 5:**
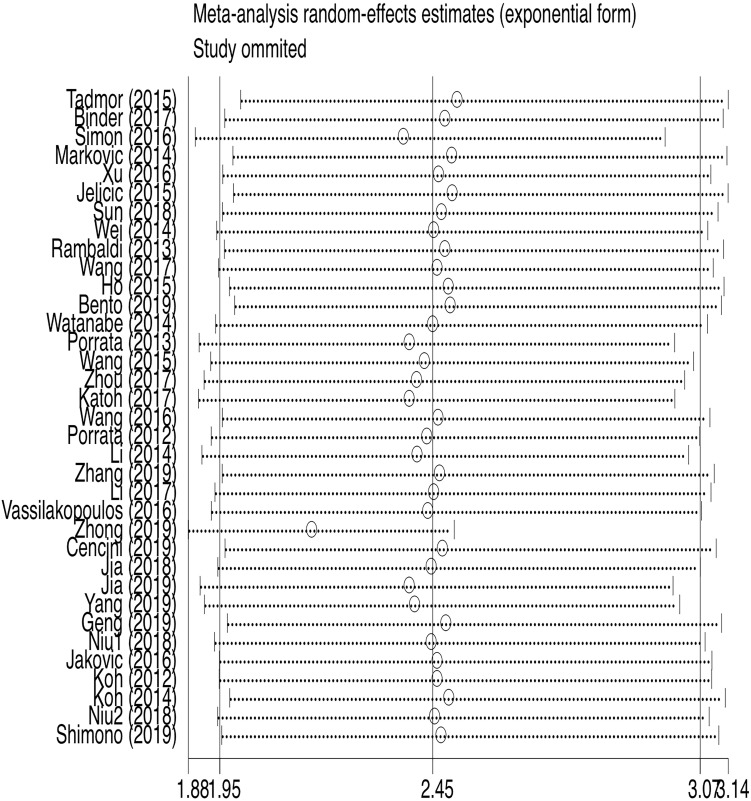
Sensitivity analysis of OS.

**Figure 6:**
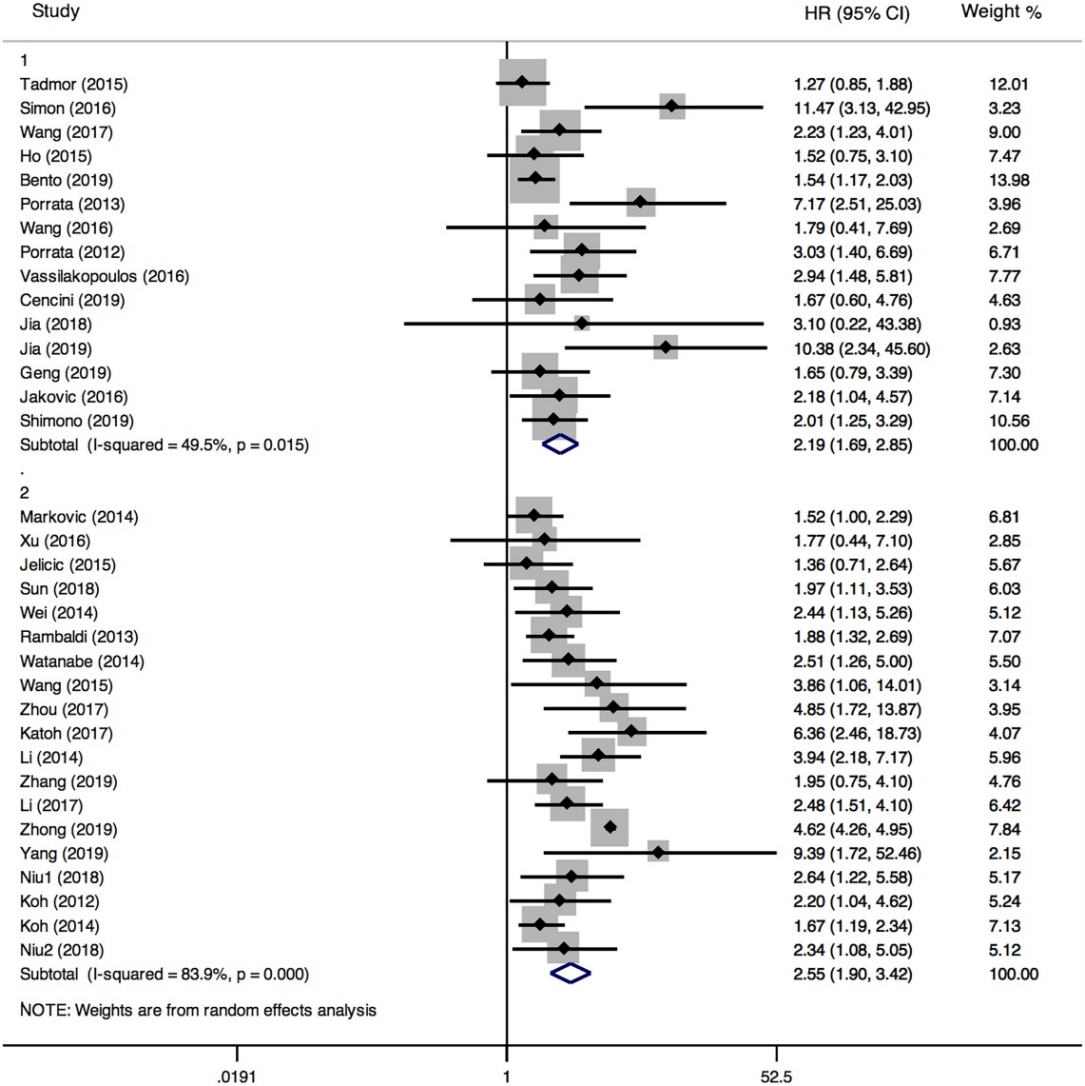
Subgroup analysis of OS according to LMR cut-off values (1: LMR≤2.5, 2: LMR>2.5).

35 studies provided relevant HRs of OS. The random-effects model was employed. The outcome demonstrated that low LMR was associated with an inferior overall survival rate, and the result was statistically significant (HR=2.45, 95%CI 1.95-3.08; *I*²=84.5%, *P*<0.000, Fig. 4). The sensitivity analysis (Fig. 5) revealed that the study Zhong (2019) had an impact on the heterogeneity of OS, and the pooled HR was 2.17 (95%CI 1.88-2.50) after excluding this article. Heterogeneity decreased from 84.5% to 36.3%, while the negative correlation between LMR and OS still existed. Subgroup analysis according to sample size, country, publication year, median age, LMR cutoff, and rituximab showed that low LMR was associated with inferior OS in each subgroup (Table 2). Subgroup analysis based on LMR cutoff value showed that the difference in prognostic significance between the two groups increased, as the LMR cutoff value increased (Fig. 6).

### Progression-free survival

**Figure 7:**
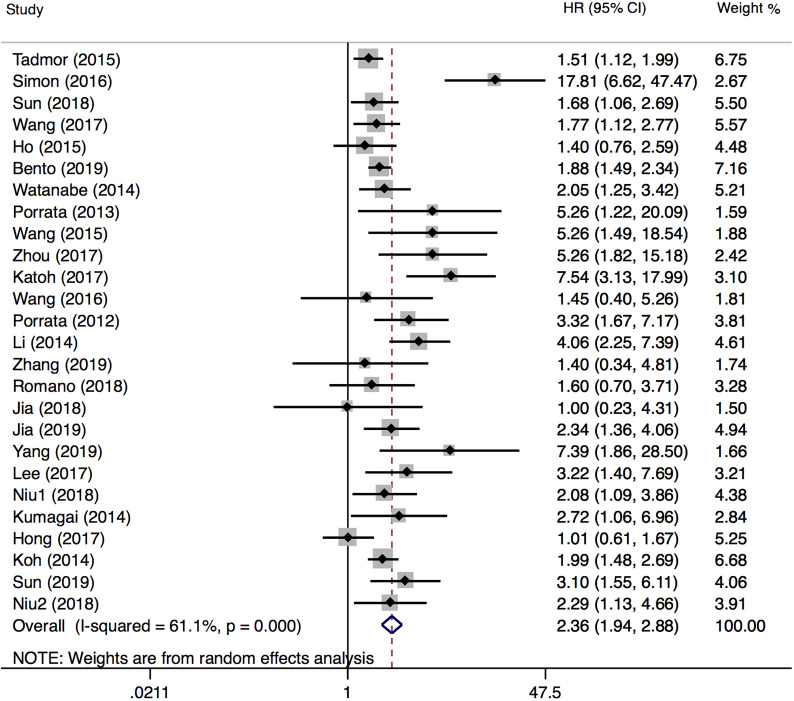
Forest plot comparing PFS between low-LMR and high-LMR groups.

**Figure 8:**
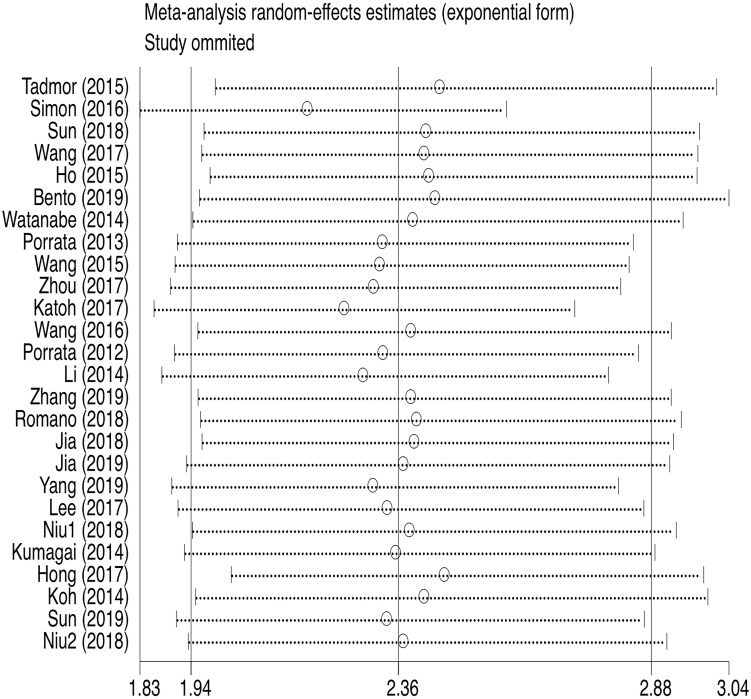
Sensitivity analysis of PFS.

PFS was reported in 26 articles. The result showed that PFS in the low LMR group was significantly poor (HR=2.36, 95%CI 1.94-2.88; *I*²=61.1%, *P*<0.000, Fig. 7). Sensitivity analysis (Fig. 8) demonstrated that the article Simon (2016) influenced the heterogeneity of PFS. After excluding this article, pooled HR became 2.18 (95% CI = 1.83-2.58). Heterogeneity decreased from 61.1% to 47.2%, but the negative correlation between LMR and PFS did not been destroyed. In addition, subgroup analysis showed that compared with the high-LMR group, PFS in the low-LMR group was poorer in each subgroup (Table 2).

### Prognostic value of LMR in subtypes of lymphoma

35 studies assessed the prognostic value of LMR on overall survival, 8 reporting on HL, 26 reporting on NHL, and 18 reporting on DLBCL. Pooled results showed that lower LMR was significantly associated with poor OS in HL (HR=3.17, 95%CI 1.89-5.30), NHL (HR=2.32, 95%CI 1.78-3.02), and DLBCL (HR=2.31, 95%CI 1.66-3.22). Detailed information was shown in Table 3. HL patients had the most obvious difference in OS between the two groups. As for PFS, 26 articles comprised this outcome, 6 analyzing HL, 20 analyzing NHL, of which 12 articles analyzed DLBCL. The results indicated that PFS was poor in the low LMR group in these subtypes.

### Association between LMR and clinicopathological characteristics of lymphoma

Relevant articles were enrolled to analyze the association between LMR and six clinicopathological features of lymphoma (Table 4). The results indicated that bone marrow involvement and pathological types were not associated with LMR. 15 studies were chosen to assess the association between LMR and B symptoms, and the combined OR showed that the low LMR group was prone to B symptoms (OR=2.13, 95%CI 1.61-2.82). The relationship between LMR and IPI score was analyzed in 13 studies. The result showed that patients with IPI scores higher than 3 were more likely to appear in the low LMR group (OR=0.40, 95%CI 0.33-0.48). 17 studies evaluated the association between low LMR and LDH, suggesting a negative correlation between LMR and LDH (OR=2.26, 95%CI 1.66-3.09). 20 studies assessed the relationship between low LMR and tumor stage, and the pooled result demonstrated that the tumor stage was relatively high in the low LMR group (OR=0.41, 95%CI 0.36-0.46).

## Discussion

Peripheral blood lymphocyte count is considered as an indicator of the host immunity. The lymphocytes play an important role in immune surveillance and defense system against tumor. The CD8+ T cells are able to recognize and eliminate tumor cells mainly through perforin and granzyme B pathways. The CD4+ Th cells modulate tumor microenvironment by secreting cytokines such as IFN-γ, TGF-β, IL-4, IL-5, and IL-6. The regulatory T cells suppress immune activation and autoimmunity. Carreras et al. found that the reduction of Treg cells is associated with tumor recurrence, transformation, and highly invasive histology 13, which remains controversial in other studies 14. In general, lymphocytosis is associated with a favorable prognosis in patients with cancer.

The monocytes are released from the bone marrow into the blood, and then migrate into peripheral tissues where monocytes differentiate into macrophages. Activated macrophages are categorized to two types, i.e., M1 and M2 macrophages. M1 macrophages have anti-tumor functions, whereas TAMs, which resemble M2 macrophages, express high levels of anti-inflammatory cytokines, angiogenic factors and metalloproteinases to promote cancer progression 15. Steidl et al. analyzed the TME in 130 classic HLs and showed that, increased number of TAMs was significantly associated with poor OS, and its prediction power was better than conventional IPI score 3. Li et al. found that AMC positively correlated with TAM in DLBCL patients treated with rituximab, and poor survival outcomes were observed in of patients with high AMC and TAM 16.

Our meta-analysis included a total of 10446 patients from 40 studies, and explored the prognostic significance of LMR on lymphoma and its subtypes. The reduced LMR are known to adversely affect OS and PFS in patients with lymphoma. The prognostic significance did not diminish in further subgroup analysis according to LMR cut-off value, sample size, country, publication year, median age, and rituximab, suggesting that peripheral blood LMR is a reliable prognostic marker. Moreover, the analysis of LMR and clinicopathological characteristics revealed that low LMR was associated with higher LDH, IPI score, and tumor stage. LDH is indicative of lymphoma burden, and a high IPI score and advanced tumor stage correlate with poor prognosis. No significant association was found between LMR and bone marrow involvement and histological subtypes of lymphoma. A recent meta-analysis included 8 studies with a total of 3319 patients with HL, and suggested that low LMR was associated with poor OS and PFS 17. Xia et al. analyzed 12 studies with 5,021 DLBCL patients, and similarly, they found that low LMR has poor prognostic implication for DLBCL 18. In this study, we updated the clinical data and analyzed the prognostic value of LMR in several lymphoma subtypes.

Cutoff values of LMR were variable among the included 40 studies, and most LMRs ranged from 2.0 to 3.0. Subgroup analysis based on cutoff values demonstrated that the differences in OS and PFS between the low- and high-LMR groups were more significant when the cutoff value was higher than 2.5. LMR cut-off values were usually 1.1~2.9 in HL, and 1.6~4.0 in DLBCL. The LMR cutoffs were calculated using the ROC curve in most studies, while median LMR or previously-reported value was selected in other studies. To better define the prognostic role of LMR, a standardized calculation of LMR cutoff is required for different lymphoma subtypes.

Although high TAM infiltration in TME is often associated with poor prognosis in lymphoma, the use of rituximab may diminish the adverse effect. For example, Canioni et al. found low macrophages was significantly associated better event-free survival in FL patients treated with CHVP-I (cyclophosphamide, doxorubicin, etoposide, prednisolone, and interferon) regimen but not in those receiving rituximab plus CHVP-I 19. We asked whether rituximab would affect the prognostic significance of LMR in lymphoma. Interestingly, a meta-analysis 18 showed that LMR could well predict OS and PFS in patients with DLBCL treated with rituximab; LMR did not affect PFS in DLBCL treated without rituximab. Since a small number of patients were included in non-RCHOP treatment group, these results need to be further verified. In our study, the patients treated with and without rituximab were assigned in 14 studies, respectively. Subgroup analysis suggested that the low LMR group had poor OS and PFS regardless of the use of rituximab, although the difference was more significant in non-rituximab groups. Thus, our analysis demonstrated that the prognostic significance of LMR for B cell lymphoma is still valid in the rituximab era.

The combinations of LMR with other prognostic assessment tools have been studied in patients with lymphoma. Simon et al. 20 suggested that in HL, the prognosis of the high LMR/PET-CT negative group was significantly better than the low LMR/PET-CT positive group, and the factors combination was more accurate than single factor in prognostic assessment. Ji et al. reported that LMR/LDH ratio had a better predictive power than LMR alone in DLBCL 21. Therefore, LMR can be used in conjunction with other prognostic tools such as PET-CT and IPI scores for better risk stratification, which could translate to individualized or precision treatment of lymphoma.

Our study has some limitations. First, the meta-analysis is based on retrospective studies rather than prospective randomized controlled trials, which might lead to publication bias. Second, not all of the included HRs have been adjusted, because covariates did not always exist. In addition, different LMR cut-off values were used in the included studies, which may lead to increased heterogeneity.

In conclusion, low LMR is associated with poor survival outcomes in lymphoma patients. As a simple and reliable prognostic marker, LMR, alone or in combination with other parameters, will be helpful for prognosis assessment. Since our results are mainly based on retrospective clinical studies, the role of LMR warrants further investigation in prospective randomized trials.

## Figures and Tables

**Table 1:** Characteristics of included studies

First author	Year	Location	Disease subtype	Sample size	R	Cut-off value	High-LMR (n)	Low-LMR (n)	OS HR (95%CI)	PFS HR (95%CI)	NOS
Tadmor 22	2015	Europe	HL	1450	-	2.1	957	493	1.27 (0.85-1.88)	1.5 (1.12-2)	8
Binder 23	2017	America	NHL+HL	390	NA	NA	NA	NA	1.860 (1.26-2.75)	NA	4
Simon 20	2016	Europe	HL	121	-	2.11	NA	NA	11.510 (3.14-42.86)	17.74 (6.61-47.57)	7
Markovic 24	2014	Europe	DLBCL	222	+	2.8	112	110	1.515 (1.003-2.288)	NA	6
Xu 25	2016	China	DLBCL	38	+/-	3.9	22	16	1.761 (0.437-7.092)	NA	7
Jelicic 26	2015	Europe	DLBCL	182	+	2.8	109	73	1.366 (0.711-2.625)	NA	4
Sun 27	2018	China	DLBCL	564	+	2.7	216	348	1.966 (1.1-3.513)	1.688 (1.062-2.684)	4
Wei 28	2014	China	DLBCL	168	+/-	2.6	87	81	2.434 (1.128-5.254)	NA	7
Rambaldi 29	2013	Europe	DLBCL	1,057	+/-	2.6	555	502	1.880 (1.32-2.7)	NA	8
Wang 30	2017	China	NKTL	379	-	2	275	104	2.230 (1.237-4.018)	1.763 (1.119-2.777)	6
Ho 31	2015	China	DLBCL	148	+	2.11	88	60	1.528 (0.751-3.111)	1.402 (0.758-2.59)	5
Bento 32	2019	Europe	DLBCL	780	+	2.25	NA	NA	1.540 (1.17-2.03)	1.87 (1.49-2.34)	5
Watanabe 33	2014	Japan	DLBCL	359	+	4	132	227	2.507 (1.255-5.007)	2.063 (1.249-3.408)	8
Porrata 34	2013	America	HL	190	-	1.1	167	23	7.140 (2.5-25)	5.26 (1.22-20)	5
Wang 35	2015	China	BL	62	+/-	2.6	38	24	3.852 (1.063-13.958)	5.252 (1.485-18.58)	7
Zhou 36	2017	China	DLBCL	173	+	3.2	73	100	4.878 (1.709-13.889)	5.236 (1.818-15.152)	6
Katoh 37	2017	Japan	DLBCL	74	+	2.6	28	46	6.380 (2.46-18.75)	7.51 (3.14-17.93)	6
Wang 38	2016	China	DLBCL	53	+	2.2	32	21	1.790 (0.41-7.69)	1.45 (0.4-5.26)	6
Porrata 39	2012	America	HL	103	NA	2.1	75	28	3.030 (1.41-6.67)	3.33 (1.67-7.14)	7
Li 10	2014	China	DLBCL	244	+/-	3.8	96	148	3.954 (2.172-7.196)	4.071 (2.243-7.389)	7
Zhang 12	2019	China	NKTL	148	-	2.7	111	37	1.950 (0.75-4.09)	1.41 (0.34-4.8)	6
Li 40	2017	China	NKTL	264	-	2.85	166	98	2.475 (1.5-4.085)	NA	6
Vassilakopoulos 41	2016	Europe	HL	537	-	1.1	477	60	2.930 (1.47-5.81)	NA	5
Romano 42	2018	Europe	HL	180	-	2	98	82	NA	1.6 (0.7-3.7)	7
Zhong 43	2019	China	DLBCL	228	+	2.7	NA	NA	4.610 (4.25-4.97)	NA	8
Cencini 44	2019	Europe	PTCL	31	-	2.1	13	18	1.670 (0.6-4.76)	NA	4
Jia 45	2018	China	ALCL	29	-	2.5	13	16	3.090 (0.221-43.299)	1.004 (0.235-4.291)	4
Jia 46	2019	China	HL	133	-	2.5	70	63	10.360 (2.35-45.66)	2.35 (1.36-4.07)	4
Yang 47	2019	China	DLBCL	28	-	3.31	NA	NA	9.434 (1.712-52.632)	7.353 (1.859-28.571)	4
Geng 48	2019	China	DLBCL	113	NA	2.27	60	53	1.641 (0.796-3.380)	NA	4
Lee 9	2017	China	FL	88	+/-	3.2	49	39	NA	3.23 (1.41-7.69)	7
Niu1 49	2018	China	AITL	64	-	3.07	24	40	2.63 (1.22-5.56)	2.08 (1.09-3.85)	6
Jakovic 50	2016	Europe	HL	101	-	2	41	60	2.185 (1.043-4.577)	NA	5
Kumagai 8	2014	Japan	FL	99	+	4.7	23	76	NA	2.714 (1.06-6.948)	8
Koh 51	2012	Korea	HL	312	NA	2.9	158	154	2.194 (1.04-4.62)	NA	8
Hong 52	2017	Korea	DLBCL	313	+	3	155	158	NA	1.006 (0.61-1.657)	6
Koh 53	2014	Korea	DLBCL	603	+	3.04	342	261	1.663 (1.18-2.34)	1.991 (1.47-2.68)	7
Sun 54	2019	China	DLBCL	43	+/-	2.6	25	18	NA	3.083 (1.554-6.117)	6
Niu2 55	2018	China	NHL	164	+/-	3.14	78	86	2.342 (1.08-5.076)	2.299 (1.13-4.673)	6
Shimono 56	2019	Japan	DLBCL	211	+	1.6	117	94	2.021 (1.245-3.28)	NA	4

HL: Hodgkin lymphoma; NHL: non-Hodgkin lymphoma; DLBCL: diffuse large B-cell lymphoma; NKTL: NK/T cell lymphoma; FL: follicular lymphoma; BL: Burkitt lymphoma; AITL: angioimmunoblastic T cell lymphoma; PTCL: peripheral T cell lymphoma; ALCL: anaplastic large cell lymphoma; R: rituximab; +: treated with rituximab; -: treated without rituximab +/-: only part of the patients treated with rituximab; NA: not available; NOS: Newcastle-Ottawa scale; OS: overall survival PFS: progression-free survival.

**Table 2:** Subgroup analysis for the outcome of OS and PFS

	Overall survival				Progression-free survival			
	Number of studies	HR (95%CI)	Heterogeneity		Number of studies	HR (95%CI)	Heterogeneity	
			*I²* (%)	*P*			*I²* (%)	*P*
**Sample size**								
≤150	15	2.73 (1.99-3.73)	28.4	0.145	14	3.05 (2.11-4.41)	59.2	0.003
>150	20	2.28 (1.70-3.04)	90.3	0.000	12	1.92 (1.58-2.32)	48.1	0.031
**Location**								
Europe and America	12	1.93 (1.54-2.43)	51.7	0.019	6	2.79 (1.69-4.60)	81.3	0.000
Asia	23	2.65 (2.04-3.43)	75.7	0.000	20	2.28 (1.84-2.82)	48.5	0.008
**Published year**								
≤2015	13	2.00 (1.61-2.49)	44.3	0.043	9	2.28 (1.72-3.01)	52.1	0.033
>2015	22	2.67 (2.00-3.58)	82.7	0.000	17	2.42 (1.83-3.22)	66.3	0.000
**Median age**								
≤50	15	2.60 (1.99-3.40)	41.9	0.045	11	2.53 (1.71-3.73)	67.4	0.001
>50	20	2.26 (1.66-3.08)	89.2	0.000	15	2.29 (1.81-2.90)	58.3	0.002
**Rituximab**								
With	12	2.23 (1.47-3.39)	92.1	0.000	10	1.98 (1.53-2.56)	57.8	0.011
Without	13	2.74 (1.95-3.86)	53.9	0.011	10	2.47 (1.62-3.77)	69.9	0.000
**LMR cut-off value**								
≤ 2.5	15	2.19 (1.69-2.85)	49.5	0.015	11	2.15 (1.59-2.92)	65.8	0.001
>2.5	19	2.55 (1.90-3.42)	83.9	0.000	15	2.55 (1.95-3.34)	57.0	0.003

**Table 3:** Prognostic significance of low LMR in subtypes of lymphoma

	Overall survival				Progression-free survival			
	Number of studies	HR (95%CI)	Heterogeneity		Number of studies	HR (95%CI)	Heterogeneity	
			*I²* (%)	*P*			*I²* (%)	*P*
HL	8	3.17 (1.89-5.30)	69.7	0.002	6	3.12 (1.65-5.90)	81.4	0.000
NHL	26	2.32 (1.78-3.02)	85.6	0.000	20	2.04 (1.80-2.30)	49.2	0.007
DLBCL	18	2.31 (1.66-3.22)	89.6	0.000	12	2.27 (1.73-2.98)	65.7	0.001
NHL except DLBCL	7	2.36 (1.76-3.16)	0.0	0.967	7	2.10 (1.57-2.82)	0.0	0.518
NKTL	3	2.30 (1.62-3.25)	0.0	0.885	2	1.73 (1.12-2.65)	0.0	0.748
FL	NA	NA	NA	NA	2	2.98 (1.59-5.61)	0.0	0.793

HL: Hodgkin lymphoma; NHL: non- Hodgkin lymphoma; DLBCL: diffuse large B-cell lymphoma; NKTL: NK/T cell lymphoma; FL: follicular lymphoma; NA: not available.

**Table 4:** Association between LMR and clinicopathological characteristics of lymphoma

Clinicopathological characteristics	Study number	Patient number	OR (95%CI)	*P*	Heterogeneity	
					*I²* (%)	*P*
Bone marrow involvement (+ vs. -)	6	1016	1.24 (0.85-1.81)	0.268	0.0	0.724
B symptom (+ vs. -)	15	3004	2.13 (1.61-2.82)	0.000	53.2	0.008
Pathological type (GCB vs. NON-GCB)	3	249	0.69 (0.41-1.16)	0.164	0.0	0.607
IPI score (≤2 vs. >2)	13	2532	0.40 (0.33-0.48)	0.000	0.0	0.503
LDH (Elevated vs. Normal)	17	4033	2.26 (1.66-3.09)	0.000	73.4	0.000
Tumor Staging (I-II vs. III-IV)	20	4296	0.41 (0.36-0.46)	0.000	42.1	0.025

GCB: germinal center B-cell-like; IPI: international prognostic index; LDH: lactic dehydrogenase.
